# What Impact Does Accreditation Have on Workplaces? A Qualitative Study to Explore the Perceptions of Healthcare Professionals About the Process of Accreditation

**DOI:** 10.3389/fpsyg.2020.01614

**Published:** 2020-07-10

**Authors:** Amna I. Alshamsi, Louise Thomson, Angeli Santos

**Affiliations:** Division of Psychiatry and Applied Psychology, School of Medicine, University of Nottingham, Nottingham, United Kingdom

**Keywords:** accreditation, healthcare professionals, psychosocial risks, workload, psychological health

## Abstract

**Aim:**

This study seeks to explore the emerging psychosocial risks of healthcare accreditation in workplaces and understand healthcare professionals’ (HCPs) perceptions of work demands and the unexpected consequences such accreditation has created for them.

**Methods:**

Twenty-seven semi-structured interviews and four focus group discussions were conducted with a variety of HCPs, including doctors, nurses, pharmacists, and allied health professionals. The study was conducted in three public hospitals and a network of primary healthcare centers in the United Arab Emirates. Interviews and focus group discussions were transcribed and analyzed using a theoretical thematic analysis approach.

**Results:**

The results showed that a number of psychosocial risks were prevalent during the course of accreditation. HCPs faced increased work demands during such a process, including increased working hours, increased working pace, perceived time pressure, and conflicting information. Such demands were perceived to influence not only their health but also their families as well as patients’ care. In contrast, teamwork and coworker support were vital to mitigate the effect of such demands.

**Implications:**

This study identified emerging risks during the process of accreditation. The findings show that the process of accreditation increases work-related risks before the inspection visit. These findings have significant implications for understanding how accreditation processes increase psychosocial risks; they also consolidate the idea that appropriate systems and support for HCPs should be a priority when planning for accreditation.

## Introduction

The concept of continuous quality improvement (CQI) has inspired the growth of accreditation programs in the healthcare sector (Shortell et al., 1998), which aims to acknowledge healthcare organizations publicly and to encourage them to improve the quality of care provided to patients. While the impact and outcome of healthcare accreditation remain debatable, the growth of such programs has accelerated significantly over the past decades (Nicklin et al., 2017). In addition, over 70 countries have employed such accreditation programs in their healthcare organizations, including developing countries such as the United Arab Emirates (Greenfield and Braithwaite, 2008; Devkaran and O’Farrell, 2015; Greenfield et al., 2019). While healthcare accreditation might be appealing to managers and stakeholders, many have argued that accreditation is a demanding activity, which increases workload and stress levels among workers (Touati and Pomey, 2009; Elkins et al., 2010; El-Jardali et al., 2014; Kousgaard et al., 2019).

The nature of accreditation is to assess the performance of healthcare facilities through an external inspection process, using a defined set of standards. When comparing accredited and non-accredited ones, accreditation has been found to support the promotion of patients’ health and safety (Shaw et al., 2010), improve the quality of healthcare services (Greenfield and Braithwaite, 2008; O’Beirne et al., 2013; Shaw et al., 2014; Bogh et al., 2016), encourage organizational change (Kousgaard et al., 2019), and allow professional development (Greenfield and Braithwaite, 2008). Furthermore, previous research has shown that accreditation can have positive effects on the quality of healthcare management and leadership (El-Jardali et al., 2014). However, a direct impact on clinical practices has not been explored in previous studies (Shaw et al., 2014; Brubakk et al., 2015). The process of accreditation has been found to increase workload, stress levels, and use of resources (Brubakk et al., 2015); In addition, little attention has been given to the consequences of mandatory accreditation on workers’ psychological health. Touati and Pomey (2009) have compared the process of optional accreditation in Canada with that of mandatory accreditation in France. They observed differences in the philosophy of applying accreditation. These differences suggest that optional accreditation enables continuity of care, while mandatory accreditation scrutinizes the delivery of care (Touati and Pomey, 2009, pp. 156–165). Moreover, recent studies have examined the effects of workload linked to the accreditation process on reduced care of patients (Ho et al., 2014; Bogh et al., 2018). Bogh et al. (2018) described how doctors and nurses were distracted by paperwork that influenced their time with patients.

Recently, many features of contemporary work have emerged, such as demographic shift, advanced technologies, task shifting, and outsourcing, which challenge organizations and increase the progression of psychosocial hazards (Zadow and Dollard, 2016). Changes in healthcare organizations can introduce new psychosocial risks, and the process of accreditation can be one of these changes. Since psychosocial risks are common in healthcare services, these risks may include work-related stress, role conflict, inadequate social support, staff shortages, work shifts, and attacks from patients (Lipscomb, 2017). Although psychosocial risks are frequently changing, such risks could put workers’ health in danger, varying from mental, social, to physical health problems. Psychosocial risks are the interactions between work and management features on the one hand, and employees’ skills and competencies on the other (ILO, 1986). Such interactions have the potential to cause physiological and psychological harm to employees (WHO, 2010). Therefore, the importance and originality of this study is that it explores whether such risks arise during the course of accreditation. While previous research on accreditation has tended to focus on promoting change and developing safety skills, such research fails to identify the emerging psychosocial risks of healthcare accreditation in workplaces and its impact on HCPs’ health as well as patients’ care. Therefore, this study aimed to investigate occupational hazards, which include increased work demands that could put workers’ health in danger, and cause mental, social, and physical health problems.

Bakker and Demerouti (2007) introduced Job Demands-Recourses (JD-R) as a contemporary model of work-related stress. They suggest that high job demand causes distress related to persistent physical, psychological, and social efforts, which are in turn linked to both psychological and physiological harm. Job resources, on the other hand, motivate workers to achieve work objectives, lower the effects of job demand, and enhance employees’ learning and development of work-related skills (Bakker et al., 2003a, b). The JD-R model links psychosocial risks, including workload, role ambiguity, and role conflict to health-related outcomes such as burnout and stress. So far, very little attention has been paid to the emerging job demands in the process of accreditation; hence, qualitative research can play a significant role in identifying the types of risks that are specific to this process. Healthcare organizations are changing regularly, and many developed countries have recognized psychosocial risks in the workplace; however, such risks can be found in developing countries as well due to globalization and changes in work aspects (Kortum et al., 2011).

While some published qualitative studies have focused on staff views on accreditation as an improvement process, to our knowledge, far too little attention has been paid to the emerging psychosocial risks during the course of the accreditation. For this reason, this study adopted a qualitative design using a theoretical thematic analysis (TA) approach to explore these risks (Gale et al., 2013). The study aims to capture the unique demands of accreditation and to develop a clear understanding of the psychosocial risks that are associated with the accreditation process. In addition, the study uses the JD-R model to ensure a better understanding of the themes developed in the study. Although a TA method does not follow a particular epistemological position (Braun and Clarke, 2006), the study attempts to identify apparent psychosocial risks and to highlight the importance of resources that mitigate the impact of these risks. Therefore, a theoretical framework offers an effective way to categorize and develop themes that explain common outcomes related to the accreditation process.

## Aims

The main purpose of this investigation is to explore how the requirements of accreditation influence the work environment in healthcare organizations. In particular, it aims to answer the following research questions:

(1) What are the psychosocial risks perceived by healthcare professionals (HCPs) during the course of accreditation?
(2) What type of resources were available to mitigate the negative impact of accreditation and support HCPs through the accreditation process?

## Materials and Methods

### Design/Methodology

The United Arab Emirates (UAE) is a rapidly growing country consisting of seven emirates: Abu Dhabi, Dubai, Ajman, Ras Al Khaimah, Umm Al Quwain, Fujairah, and Sharjah. The rapid growth of the country has been observed in its population and economy, which has influenced the healthcare system positively. The Ministry of Health and Prevention is a government-funded healthcare system that oversees more than 17 hospitals and 72 primary healthcare centers distributed across the country. These facilities provide comprehensive and sustainable health services for individuals and society. A qualitative research design was employed to provide an in-depth understanding of the psychosocial work environment during the accreditation process. Hence, the study interviewed 27 HCPs from three hospitals and conducted four focus group discussions with HCPs from sixteen primary healthcare centers after they had achieved their accreditation certificates.

The study used interviews and focus group discussions to understand the perceptions of HCPs from different working environments. Interviews aimed to explore the variation of work aspects in hospital settings, while focus groups attempted to expand the knowledge developed from interviews through discussions with HCPs from primary healthcare settings. Interviews and focus groups were conducted in two different locations, the findings, however, were similar and provided comprehensive interpretations of the data. By applying both methods, the study attempted to address the limitations often discussed in quantitative research, which ignore participants’ experiences and fail to provide a clear picture of their views (Carr, 1994). Although quantitative tools are available to test various types of psychosocial risks and their association with the experienced demands at work, these assessment tools are generic and fail to explore the unique risks associated with the process of accreditation in healthcare facilities. Furthermore, such tools fail to provide detailed reflection of the shared perceptions of HCPs regarding the increased risks during the course of accreditation. Therefore, the contribution of applying a qualitative study is to highlight the authentic descriptions of HCPs during the process of accreditation in different healthcare settings and to explore the psychosocial risks that emerged during this process. Thereby, the study aimed to convey HCPs’ perceptions and experiences regarding accreditation by adding rich and diverse quotes that enable readers to understand their experience (Smith, 2018).

### Ethical Approval

Prior to commencing the study, ethical permissions were obtained from the Division of Psychiatry and Applied Psychology Research Ethics Sub-Committee at the University of Nottingham (i.e., Reference Number - 0236) and the Ethical Committee of the Ministry of Health and Prevention in the United Arab Emirates (MOHP/REC-40/2018).

### Selecting Participants

To understand healthcare workers’ perceptions about the process of accreditation, the study deliberately selected three hospitals and a network of primary healthcare centers, which had gone through the process of accreditation three to twelve weeks prior to data collection. Although the approach of selecting facilities could increase the reliability of the developed findings, the study could not prevent selection bias of participants. Participants were initially approached by emails sent to the directors of the selected facilities describing the nature and purpose of the study and requesting that invitation emails be sent to HCPs. Emails were sent to all workers in both English and Arabic. To maximize the number of participants in the study, the researcher approached HCPs in two ways. First, the first author waited for participants to reply to the invitation emails. HCPs who had replied to the invitation emails were not enough to achieve data saturation; therefore, the interviewer also invited HCPs at their facilities because of her approved access to the selected hospitals. It was made clear to all participants that they were under no pressure to participate in the study; hence, only HCPs who volunteered were interviewed. The primary inclusion criteria were opened to HCPs who provide services to patients to capture broad perceptions and to understand the changes that influenced the delivery of these services. Physicians, nurses, nurse assistants, pharmacists, laboratory technicians, and radiologists were recruited. Twenty-seven semi-structured interviews and four focus group discussions were conducted with a variety of HCPs, including 16 doctors, 20 nurses, 3 pharmacists, 5 allied health professionals, and 5 administrative workers. Supplementary Table 1 presents a descriptive number of participants in interviews and focus group discussions. A wide variety of HCPs was chosen to obtain a representative sample considering the resources available to conduct this research. The study attempted to select a representative sample at healthcare organizations level, hence, a homogeneous sample was achieved by recruiting participants who have never experienced accreditation. None of the healthcare organizations in this study have ever experienced accreditation. Therefore, experiencing accreditation for the first time was expected to change the working environment and to influence the psychological health of HCPs working in these organizations.

## Data Collection

Twenty-seven participants were interviewed, and four focus group discussions were conducted. Participants from interviews and focus groups were given information sheets that described the purpose of the study. After reading the information sheets, participants were asked to sign a consent form prior to commencing the interviews and discussions. Interviews and focus group discussions were recorded using a digital recorder and were uploaded to a password-protected database and erased from the recorder. Interviews were conducted first in hospitals within three to six weeks after achieving accreditation. The time was selected to reduce the effect of errors related to the recall period (Warner et al., 2005) and to increase the reliability of the study data. In addition, the study selected hospitals that had achieved accreditation for the first time to increase the strength of the data and avoid bias related to recalling the psychosocial risks influenced by the process of accreditation. The study used 12 semi-structured questions focusing on the emotional, managerial, and professional impacts of the accreditation process before, during, and after the inspection visit. These questions are presented in Supplementary Table 2. The questions were structured according to the job demands-resources (JD-R) model (Demerouti et al., 2001) to identify aspects of job demands and job resources during the course of accreditation. Furthermore, questions probed work-related risks associated with such processes, including increased work demands and work pace, in addition to the ways in which these facilities recognized and managed such risks. The time taken for the interviews ranged between 20 and 60 min.

**FIGURE 1:**
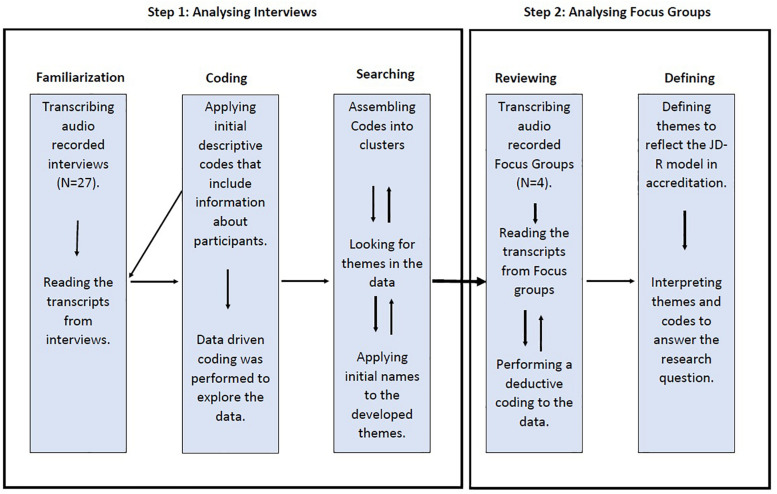
The process of analyzing interviews and focus group discussions.

Focus group discussions, which followed a confirmatory approach to support findings, were conducted after the interviews. The nature of applying such data collection methods allowed participants to debate and expand the discussion on the process of accreditation (Smith, 2015). Therefore, four focus group discussions consisting of 22 participants (Supplementary Table 2) were delivered in a different setting, that is, primary healthcare centers, to understand and broaden the response rates from a different perspective. Group discussions allowed for a dynamic debate between members and enhanced the interpretation of findings developed from interviews. The discussions were stratified according to participants’ occupations: the first focus group was open to nurses and nurse assistants only, the second group targeted physicians and dentists, the third group aimed to talk to allied professionals, including pharmacists, radiologists, and laboratory technicians, and the fourth group was open to managers and administrators. By stratifying the focus groups, the study provided a safe space to allow participants to share their knowledge and to feel comfortable when providing their honest views in front of others. The time taken for focus group discussions ranged from 23 to 50 min. Figure 1 illustrates the analytic trail of coding for both interviews and focus group discussions.

### Data Analysis

Themes were developed through a systematic search for similarities in the transcripts that could explain patterns of changes in the work environment during the accreditation process. The TA approach of Braun and Clarke (2006) was employed to analyze and capture the uniqueness of demands and resources perceived during the process of accreditation. Considering its wide and flexible approach, TA was used to develop a better understanding of the psychosocial risks that go hand-in-hand with the accreditation process. While such an approach is not guided by a theoretical framework, TA can follow both realist and relativist assumptions, and can range from a simple descriptive approach to a more complex approach that reflects deeper meaning in the data (Smith, 2015). Although TA does not follow a particular epistemological approach (Gale et al., 2013), it is essential to understand the rationale behind applying such an approach to analyze data. This study followed a theoretical approach because it seeks to develop a thorough understanding of different aspects perceived by HCPs with regard to the JD-R model. Since the research questions were created based on a well-known model, that is, JD-R, a deductive TA approach was used to analyze the data. Smith and McGannon (2018) addressed the difficulty of analyzing data without prior knowledge of a theory. Therefore, themes were established through a systematic search for similarities in the transcripts that could explain patterns of changes in the work environment during the accreditation process and link them to the JD-R model.

The study followed a deductive TA approach that used the JD-R model to develop codes and themes that appeared in the data (Braun and Clarke, 2013). Such approach was used to present the relationship between codes and to create a conclusion that reflects the JD-R model. Although the study used a deductive approach to code data, the analysis of interviews and focus group discussions moved beyond the exact meaning of codes to explain changes in work aspects with the process of accreditation. In addition, the homogeneity of the sample size provided more focused codes and themes with regard to participants’ views in accreditation. Because the study used a large sample size, interviews were first recorded and transcribed to develop an initial understanding of HCP perceptions about the process of accreditation. Focus group discussions were then conducted for further elaborations on such perceptions. Interviews and focus group discussions were transcribed and checked for accuracy. Identification numbers were used to recognize participants, for example, P001 for the first participant in interviews and FG001 for the first focus group session. The qualitative software NVivo 12 was used to manage the data and facilitate the coding process.

Braun and Clarke (2006) used six steps to thematically analyze qualitative data. Themes were developed through a systematic search for similarities in the transcripts that could explain patterns of changes in the work environment during the accreditation process. Once interviews and focus groups were transcribed, it was necessary for the first author to be familiar with the transcripts. Familiarization involved frequent readings of the transcripts to engage in in-depth knowledge of the data set. Then, initial codes were developed using a descriptive coding technique (Saldaña, 2016). The first authors coded the interviews’ transcripts to look for similar patterns in participants’ perceptions with the accreditation process. Initial codes were descriptive and classified participants regarding their information such as gender, location of their workplace, previous experience with accreditation, and years of experience. Then, the study adopted a deductive coding technique to code responses that could answer research questions (Smith, 2015). Codes were then reviewed and categorized into clusters where similar codes were grouped into two distinctive segments that include demands and resources. To assess the reliability of the analysis, the second author reviewed and agreed on the validity of the developed codes. Although using a team member to check the codes is widely used, such inspection cannot produce codes or themes without linking them to a prior theory (Smith and McGannon, 2018). Initial themes were created by clustering codes with similar concepts that reflect the JD-R model.

Themes were then created to reflect the relationship between the different research questions. Weaker themes were grouped together to form sub-themes. Themes were then reviewed by the rest of the authors to refine and distinguish different themes from sub-themes. The review process consisted of reading the entire codes and checking the coherency and relevance of the developed themes. Sub-themes were named and identified according to the scope of psychosocial risks in healthcare accreditation and their influence on HCPs. Themes, however, were named to reflect the JD-R model in accreditation. These themes were then supported by data gathered from focus group discussions to confirm findings from interviews. Despite the differences in the working context, focus group discussions reinforced the elaboration of themes constructed from the interviews. Furthermore, focus group findings confirmed the similarity of changes in the working environments influenced by the process of accreditation despite the differences in contexts. A deductive TA generated two key themes that described different work experiences and outcomes of the accreditation process.

### Validity and Credibility of Codes

Considering the open approach of qualitative studies, it is vital to develop sets of standards to check the validity of such research. Therefore, this study used the same terms used by participants to maintain the credibility of codes and to reflect the experience of accreditation as recommended by Whittemore et al. (2001). Furthermore, a valid and grounded interpretation of participants’ own words was necessary to maintain the integrity and representation of the results (Smith, 2018). To assess the robustness of the themes, the first author, who conducted the interviews and focus group discussions, coded the transcribed data. The second and third authors then reviewed and checked these codes to assess the accuracy and credibility of the developed themes.

## Results

The TA generated two main themes to describe the accreditation process that could pose psychosocial risks in healthcare facilities and considered participants’ perceptions of the experience. Supplementary Table 3 summarizes themes and codes of the theoretical TA. These themes reflected the two main constructs of the JD-R model and included the following:

(1) Challenging factors in the process of accreditation.
(2) Enablers to achieve accreditation.

Themes are further explained in sub-themes and quotes to describe key findings in interviews and focus group discussions. To preserve the meaning of participants’ responses, the researcher corrected grammatical errors in participants’ quotes where necessary and removed speech fillers, such as “you know” and “okay.” In addition, words were added in square brackets to clarify meanings in quotes and maintain anonymity of names disclosed by participants.

### Theme 1: Challenging Factors in the Process of Accreditation

This theme focused on the preparation phase and challenges that participants faced while preparing for accreditation. The experienced work demands described in this theme extended from the beginning of the preparation phase, which varied from 6 months to 3 years, depending on the size and complexity of hospitals and primary healthcare centers, until the visit of the external inspectors. Although the preparation phase was extended from 6 months to 3 years, the majority of the participants felt the pressure of the accreditation process predominantly 1 month prior to the arrival of the inspectors. Four sub-themes were identified in this theme, and included focused efforts on administrative work, observed work-related risks, managements’ role during accreditation, and perceived pressure from accreditation demands.

#### Focused Efforts on Administrative “Work”

To understand and prepare for the newly adopted process, HCPs were involved in different administrative activities. These activities involved attending training sessions, reading policies, and participating in frequent meetings. At first, HCPs were eager and willing to participate in such activities, and they looked forward to the change accreditation would bring to their facilities. In all cases, participants stated that they went through a series of training sessions, which aimed to clarify the newly developed policies, procedures, and practices, for example, surgical safety procedures, infection control protocols, and fire safety practices. Although training programs were frequent, participants argued that they had to attend either before or after their working hours because of their busy work schedules.

P004: For us it is not, [that] we cannot leave the department, and we have to adapt ourselves to do the courses, after our duty, or before our duty [hours]. If the course is at 10 am and my duty at 7 pm, I will come at 10 to finish the course by 1 pm, then I will go back home, then I will come back at 7 pm.

In addition, some of the HCPs were involved in improvement projects aimed at enhancing the quality of services in their departments. Such projects are called quality improvement projects and are measured by performance indicators. Due to their involvement in such projects, HCPs had to respond to frequent calls and attend different meetings to clarify and update the management on the status of their projects. Therefore, such activities were found to be time-consuming and to take away valuable time that could have been devoted on patients:

P012: A lot of time had been consumed because every week there would be one hour or two [hours] I had to shell out either from my clinic or from the operating room. So I had to be on my toes knowing that, yes, today I might be called many times for a particular meeting to clarify things.

An apparent focus during the preparation phase was the emphasis on documenting patients’ medical records as well as monitoring the improvement of the documents to a predefined goal. A common view among participants was that they focused mainly on completing patients’ medical records. Hence, HCPs concentrated their efforts on improving these records before the inspection process. While all agreed that documentation was a safety practice for both patients and workers, some HCPs argued that they were engaged in the documentation task, which, as they perceived, reduced the actual care of patients. One individual stated that the process of documentation put them in front of computers, which could indicate less engagement with patients and more focus on producing perfect records.

P013: So with this accreditation system, it focuses on documentation, timing of staff and putting orders in the system, and all this stuff. Thus, it pulled us away from patients and put us in front of the computer. This is what had happened for nurses, for lab technicians, for doctors, for [emergency] physicians, for everyone.

Not only had focusing on documentation influenced patient care, but attending meetings with direct managers or other staff to discuss the status of the preparations, was also perceived to have influenced the delivery of care. One participant stated:

P017: So appointments were canceled and appointments were postponed. Delays in inpatient services. It was seen and provided, but it was delayed more than usual or at the time that it was supposed to be given.

#### Observed Work-Related Risks

One of the risks identified during the preparation for accreditation was the additional working hours that HCPs had to spend at work. A possible explanation for spending additional hours at work could be that HCPs felt responsible for achieving such accreditation. The majority of participants felt the need to sacrifice their leisure time to achieve the accreditation certificate. It was not possible for them to finish work demands within the official working hours, and spending additional hours at work was seen as essential in order to receive the accreditation. For others, they decided to work additional hours because they could not compromise on mixing patients’ care with accreditation demands:

P009: We worked hard. We used to leave the hospital so late, after finishing our duty, we used to leave late from work.

In addition, the staff shortage was a vital reason why participants felt overloaded with work. Due to the staff shortage, workers were challenged to finish the requirements and take care of their patients at the same time.

P024: …if they [would] provide us [with] more staff, it would be fine. We [could have done] the documentation and patients’ care all together, but because of the shortage of staff we [were] really having a hard time doing this.

While some HCPs worked for additional hours to finish their work, others had to complete their work from home. In contrast, few interviewees were able to manage the requirements without the need to spend extra hours working.

P012: I never had to stretch beyond my limits to stay back [at the hospital] two or three hours just for this work.

Participants explained as well how their work pace had increased to complete and meet the standards of accreditation before the inspectors’ visits. HCPs might have worked faster because of the late implementation of new adopted standards and policies. There are two possible explanations for the late execution of these standards. First, accreditation is a change process, and change requires sufficient time from workers to accept and adapt to the new standards. It is possible that resistance to change lead to the delay, as participants were accustomed to previous practices and found it difficult to adapt to the new ones:

FG003: For me, I faced difficulties. Some of the staff did not accept these changes; there was some sort of negligence from the staff. Therefore, when we released the new [policies], we used to chase them. So, we used to teach them and inform them what to do… and for me, it was a challenge to come and ask them to work in a certain manner in a few months. Until now, I face the same challenge to change their mentality, [to ask them] to read and be updated about the policies. Yeah, there were difficulties.

The second explanation for the late execution of standards could be the late arrival of resources, such as equipment and staff. Although resources were provided, these resources arrived late and challenged workers to complete the required demands within a limited time and with limited resources:

FG001: They have to give [us] the resources and the staff, whatever it is; staff are also resources. All the things to be in [our] hands, then [we can] start working. Therefore, it will not be tense and will go smoothly. This will not waste our time. Therefore, we can reach the goal very fast instead of wasting [our] time.

Due to limited resources, HCPs felt uncomfortable and overwhelmed by the requirements of accreditation, including increased work demands prior to the arrival of inspectors. Although work requirements were manageable, the time needed to fulfill the requirements created a sense of discomfort among HCPs.

P010: I mean, squeezing us in this small period [of time] to do all the things. This was the worst thing at that time.

A common view among participants was the stressful feelings that went hand-in-hand with the preparation process, which was caused by the increased pace of work. Work pace describes the speed of work, and the pace HCPs maintained to organize their workplace before the arrival of inspectors. Participants felt they were pushed in a limited time to complete the required tasks before accreditation. Increased work pace was expressed through different statements about the speed of work. Many participants used the phrase “*we were running*” or “*we have to run*” to describe the pace of work prior to the arrival of inspectors.

P017: things were just announced at the last moment. And [we] had to rush through it to finish and [we] were not sure if it was right or wrong. There were things that had to be rushed and finished in the morning that [inspectors] were here. Therefore, [we] had to run from one office to another.

Furthermore, conflicting information and frequent changes in tasks requested from HCPs during the accreditation process were found to be wasteful. HCPs were obligated to repeat or update certain tasks before the inspection date. Such conflict was created confusion and uncertainty about fulfilling certain requirements. Information that was perceived as conflicting was said to be given at the last minute and was a source of considerable frustration.

FG002: if things were clear from the beginning, it would not be easier, but better for the preparation. I mean, our time was wasted because we were repeating things. Because everyone was saying something different.

#### Perceived Pressure From Accreditation Demands

This sub-theme refers to the pressure felt by HCPs to manage the demands of accreditation. It describes the impact of additional challenges placed upon HCPs during the preparation phase, which influenced participants’ health and personal lives. A number of participants indicated that they had health problems during the preparation process for accreditation. HCPs reported high levels of stress due to their limited knowledge and skills related to the requirements of the process. Furthermore, HCPs faced additional work demands, such as working on files and paperwork, which intensified their work-related stress. One of the participants said:

P020: At first nervous. Overly stressed – I can say – and a lot of work, a lot of paperwork, we [had] to read, we [had] to understand what [we were] reading, [we had] to apply it. I mean before accreditation

While the majority agreed that preparing for accreditation was a stressful experience, a minority noticed changes in their health. These problems included behavioral, psychological, and medical problems. Due to the increased workload and time pressure, HCPs noticed a change in their eating habits, such as increased consumption of carbohydrates. Some of the participants reported weight loss, while others reported weight gain. Altered eating behavior could have contributed to weight fluctuations during the process of accreditation. Anger issues were observed by participants during this process. One of the HCPs said that she would prefer to have enough time to prepare instead of being angry and snapping at others, which could indicate problems induced by increased work demands. Other health problems such as musculoskeletal problems, digestive problems, and sleep disturbances were observed before the inspection process.

Psychological consequences, other than stress and anxiety, were recognized during the preparation process. A few HCPs stated that they had to seek psychological consultation and had taken prescription medication during the preparation phase. Although participants were aware of their psychological health prior to accreditation, the increased psychosocial risks during such processes had worsened their mental health.

P028: To be honest, I experienced a lot of stress. I have anxiety issues and, unfortunately, I had some, to a lesser extent, anger problems, and anger issues. So I had to go and see a psychologist for that… honestly, it started with. It started before, but when I felt that I could not handle it anymore it was like, just to say, one month prior to the actual arrival of the [inspectors].

Furthermore, preparation for accreditation had a clear impact on HCPs’ families and social lives. Participants felt that work tension had transferred to their homes as they continued working from home to manage the workload and additional tasks. HCPs faced difficulties in balancing work demands related to the accreditation process and their family time. Participants often mentioned that working additional hours took away valuable time that could have been devoted to their families, and they faced problems adjusting their working schedules to their family needs:

FG002: During the [accreditation] preparation, it was really stressful for us as physicians, for our patients, and even [for] our families.

#### Managements’ Role During Accreditation

Participants had mixed views about the role of management in handling psychosocial risks during the preparation phase. Therefore, this sub-theme identified the way managers recognized and reacted to work demands during preparation. Some participants expressed the need to have policies and systems in place to support their psychological health when meeting such demands and when experiencing time pressure. In addition, participants perceived that their managers would listen to their problems or suggestions, but they would not react to them. Hence, one of the HCPs explained why she avoided reporting work-related injuries.

P016: Actually, I feel bad that I am giving a lot of effort and I am not taking care [of] myself,…, also, on the other hand, no one will respond to that, no one will take care [of us].

Surprisingly, participants felt that their managers were stressed as well during the preparation time; therefore, they were not able to show support.

P001: I think because [my supervisor] was already busy, she was [conducting] meetings with us, trying to revise the policies with us, but nobody actually concentrated or thought about [the] psychological effect or the stress [placed]on the staff,….because we [felt] she was already stressed.

Participants were uncertain about the psychological support provided during increased work demands, and they were uncertain when asked about the support or activities that were planned to reduce their perceived pressure. Interestingly, few participants expressed their need to have a strong and firm style of management that engaged workers during the preparation phase. The HCPs felt that they would not have achieved accreditation without the pressure exerted by their managers. It was common for managers and supervisors to remind HCPs frequently about inspection visits and to get HCPs to achieve accreditation:

P004: You cannot blame anyone if you are in this stressful situation because it is something mandatory to keep the hospital working, it is required from the Ministry of Health. They have to get it, they have to push all the people in the same way, they cannot push you in different ways like they are dealing with me because we have different mentalities and different attitudes. If they will treat each one according to their mentality and attitude, it will be difficult for the higher management to finish it, so they have to be like this, but, it is on the other side, [workers] who are receiving, it will be stressful [for them].

Another challenging risk observed during the accreditation process was the lack of control over HCPs’ leave. It was mentioned by a number of participants that the management had strict rules for permitting leave prior to the arrival of the inspectors. Participants felt uneasy about not having control over their leave. In addition, HCPs felt that they were asked to work on their holidays, although they were allowed to take their leave due once they had achieved accreditation.

P012: [staff] were stressed, and a couple of them could not get their leave until the last minute. That is surprising because of the accreditation process [managers] wanted every individual to be around.

Although a fair number of participants commented on the general fear and anxiety they felt due to the uncertainty of the accreditation process, participants felt that they might be blamed if they failed to achieve the accreditation. HCPs echoed the blame culture at their facilities, and they were afraid to be held responsible if they failed to answer questions asked by inspectors. As a result, they used to spend additional hours on the requirements for accreditation. Moreover, HCPs did not want to disappoint their coworkers or supervisors during the inspection visits. Managers explicitly reminded HCPs of the blame culture to engage workers in the preparation phase.

P012: I think the one single thing that I have noticed very prominently is the anxiety and the fear that was instilled, maybe coming from top down, from management level, coming down all the way to clinicians, to everybody. “We have to get the accreditation and if we did not get an accreditation, the owners would be in this particular department.”

### Theme 2: Enablers to Achieve Accreditation

The HCPs mentioned possible factors that eased the effect of increased risks during the process of accreditation. The analysis in this study considered these factors as enablers or resources that were highlighted during the accreditation process. These resources were based on interpersonal relationships, and perceived support from coworkers and managers. This theme has two main sub-themes that include supportive approaches in accreditation and perceiving meaningful work after accreditation.

#### Supportive Approaches in Accreditation

A common view among HCPs was observed teamwork and collaboration between HCPs. Participants acknowledged group efforts while working on the requirements, and the majority felt responsible for achieving such accreditation. The HCPs felt comfortable working in teams, and their work was perceived to be easier due to the teamwork. While some participants suggested that the presence of procedural policies and full documentation of patients’ records granted them the needed support, others received strong support from coworkers during the increased workload to achieve accreditation, which eased the effect of the process and was found to create memorable moments.

P023: I sometimes mean [we] feel that [it] is too much. But the team was good. I mean there were quite a few people, you know, who were really dedicated and we worked out of fast. So, whenever you have teamwork, you feel good. I mean, sometimes even if [we] are stressed [we] just sit and have coffee together, [we] laugh and feel good about those things.

Some participants felt that their managers supported them during the increased workload to achieve accreditation. Managers were close to their subordinates during the final phase of the preparations, and a sense of collaborative teamwork was clear during this phase. The type of support managers provided was through simple words of encouragement. Support expressed by participants included open access to supervisors and managers during accreditation. Many HCPs were pleased to work directly with their managers:

P027: It is very rare to have the director and the medical director in this office here, but [during the accreditation] period we had them [here], they discussed with us the policies and highlighted certain areas. I felt like they [were] closer to us. Usually, they have the administrative part of work, and we have our clinical work. Therefore, we do not come to meet each other. Therefore, in the [accreditation] period we [worked] together. I felt that they were supportive.

The majority of participants agreed on the approach their manager took to recognize their hard work during the preparation period, by allowing the HCPs to take leave and permissions as a compensation for their additional working hours directly after achieving accreditation:

P028: Also, I would say that they have provided us later on with the public holidays because the accreditation came at the time of our national day. Therefore, we were asked to come to work on these days, which was a public holiday; thus, they compensated us with a day off.

Despite the fact that the majority agreed on the compensation of working days, some HCPs argued that their additional working hours were not fully rewarded, as the process of such compensation was not clear. Although interviewees took their time off after attaining accreditation, it was also likely that managers had requested employees to work additional hours during the preparation phase before inspectors’ visit. The reported facts seem to support the assumption that managers preferred a positive inspection process to avoid criticism (Ehlers et al., 2017). HCPs were asked to work additional hours, although they would be rewarded after accreditation with equivalent days off. In addition, participants felt close to their managers in social events that followed the announcement of their accreditation status. HCPs felt recognized when their managers thanked them for their contributions to such an achievement.

#### Meaningful Work After Accreditation

This sub-theme suggests that getting an international accreditation motivates HCPs throughout the process of accreditation. When asked about their feelings afterward, the majority of participants felt proud to achieve an international accreditation. The HCPs felt that they were part of an international community, wherein all accredited healthcare facilities speak the same language of quality and patient safety. A possible explanation for such feelings is that HCPs valued accreditation outcomes due to the effort they had put in order to achieve the accreditation, which is known as the IKEA effect (Marsh et al., 2018). Moreover, having policies and good documentation in place gave them a sense of confidence that work was more accurate and safer for both patients and staff. Others felt that due to the knowledge that they had gained, they were confident in working in any organization. HCPs noticed an increased sense of work engagement after attaining accreditation. A possible explanation for this may be that HCPs were involved and committed in the preparation process.

P020: I mean before it is just like I will come and go for [my] duty, I do something only [for the] patient’s care, I do not need to do this one, and I do not want to do this.” It is just like [to] come and go, but now, um, during the accreditation, I have to do something meaningful. I mean something meaningful in my life that I am doing because not only [it’s] for me but [also] for my colleagues [and] for the patients.

Accreditation may have contributed to an increase in the sense of confidence and work engagement among workers. However, some participants would not like to repeat the process again or be part of the preparation:

P028: I feel relieved and I do not want to go through that again.

FG001: Those [staff] that were like in a [state of] tension [during] that time, I think they do not want to do it again. They do not want to repeat that on the next [accreditation].

The analysis shows that accreditation has a positive impact on organizing aspects of work and promoting change in healthcare facilities, particularly in safety practices. The data describes the structured working environment and the way accredited facilities are operating after such achievement. HCPs’ opinions about the working environment after attaining accreditation were positive. They became aware of a more organized work environment and safety procedures when dealing with patients. In addition, unnecessary processes were removed from certain professions. For instance, nurses reported that they used to perform certain tasks that were not part of their job description, that is, storing and managing medications. HCPs perceived that accreditation accentuated their tasks and responsibilities, and they observed that accreditation created a common patient-safety language among them. It seems that having clear policies and procedures introduced a safer working environment for HCPs. Participants took note of standardized work in their workplaces, which increased their level of confidence.

P010: It is now organized. I feel it is organized, I mean, now everything in its place, and dealing even with patients, we have specific [procedures] like, right and wrong, and all these things. We have many things changed and removed from the departments, which were not needed, and not to be used.

## Discussion

Although the impact and outcome of accreditation in healthcare organizations remain debatable, many countries, including the United Arab of Emirates, mandate such assessments for a better delivery of healthcare services. This study provided a rich understanding of potential psychosocial risks associated with the accreditation processes taking place in healthcare organizations. Accreditation programs have been found to promote change, standardize work, and limit potential errors caused by different practices (Greenfield et al., 2012). Further, HCPs are motivated to work in accredited organizations, which enhance their engagement in the workplace. This study aimed to explore the emerging psychosocial risks during the course of healthcare accreditation. By using a qualitative paradigm, the study was designed to understand the emerging psychosocial demands faced by HCPs in the context of accreditation, the unexpected pressure it had placed upon them, and the resources needed to manage such increased demands.

The study used interviews and focus group discussions to understand the perceptions of HCPs from different working environments on the accreditation process. The analysis of such interviews and focus group discussions generated two main themes: *challenging factors in the process of accreditation* and *enablers to achieve accreditation*. The process of accreditation started when facilities adopted and generated new policies and procedures to promote a safer environment for patients. After this, workers received sufficient training to work in line with such policies. Initially, HCPs were excited to be part of the process; however, such excitement declined as workers approached the inspection date. With respect to the research questions, it was found that preparing for accreditation went hand-in-hand with increased psychosocial risks, such as increased job demands and work pace, conflicting information, and perceived strain. Such findings support evidence from previous observations regarding the negative impacts of accreditation, which include increased workload (Touati and Pomey, 2009; El-Jardali et al., 2014), increased stress levels (Kousgaard et al., 2019), and use of resources (Brubakk et al., 2015).

In line with the literature, this research found that HCPs who went through the accreditation process were proud and confident. HCPs felt they were knowledgeable and engaged in their workplace because accreditation focused on overlooked training areas such as managing organizational safety and emergency codes. Accreditation standardized the delivery of services to patients by creating a shared patient-safety language among HCPs (Bogh et al., 2018). Although the preparation phase extended from six months to three years, the majority of HCPs felt the pressure of achieving accreditation one month before the arrival of the inspectors. This pressure was due to the additional demands placed on HCPs and the late start to implement standards required to achieve such accreditation. A possible explanation for this delay could be the time needed from workers to adapt to such change. Accreditation is a change process that adds new job demands for individuals working in healthcare organizations. Previous research has suggested that organizational change is perceived as a traumatic event causing distress and disturbance among workers (Houdmont and Leka, 2010). Organizational change can create ambiguity about the role of individuals and the future of organizations.

With respect to the first research question, preparing for accreditation seems to increase work demands and workload; therefore, HCPs’ attitudes toward the process of accreditation were negative. HCPs had different responsibilities prior to the inspection visit. For example, participants defined their roles during such process as taking care of patients, familiarizing themselves with the new standards, attending different training courses, and completing patients’ records and paperwork. Such an increased workload led HCPs to work additional hours. Furthermore, HCPs observed an increased pace of work to manage such requirements. HCPs often described working during the preparation as running to complete accreditation requirements. Therefore, increased work pace is one of the most obvious findings to emerge from the analysis.

While documentation, the process of recording patient’s medical status, is an essential requirement to assess the quality and safety of healthcare services, the current study found that such requirements compromised the time spent with patients, as HCPs focused on enhancing the quality of such records. Furthermore, attending frequent meetings related to the accreditation process was found to delay the delivery of services to patients, such as canceling or rescheduling appointments. These results seem to be consistent with recent studies indicating that efforts made in preparing for accreditation are found to compromise patients’ care (Ho et al., 2014; Bogh et al., 2018). In addition, HCPs exhibited a range of health issues before the inspectors’ visits, which were attributed to the preparation process. While some had medical issues, such as musculoskeletal and digestive problems, others had behavioral problems such as sleep disturbances and anger issues. In addition, a number of HCPs noticed changes in their psychological health and the need to take medication before the assessment.

The study revealed a shared sense of fear and anxiety among HCPs during the inspection process. At first, HCPs were anxious about the uncertainty of the process of accreditation and the type of questions the inspectors might ask them. Therefore, HCPs tried to avoid such encounter by changing their shift duties or taking permissions, although taking leaves were not allowed before the inspectors’ visit. Unexpectedly, management restricted any kind of leave or permissions during the visit of the inspectors. Additionally, HCPs felt that they would bear the responsibility for not achieving accreditation as managers explicitly reminded workers to be prepared; otherwise, HCPs would be held accountable for not achieving accreditation. Due to the blame culture, HCPs worked additional hours to avoid such responsibility. In contrast, when healthcare workers met the inspectors, they felt comfortable and relaxed. They sensed that the purpose of the visit was to ensure a safe environment for patients.

The second question in this study sought to identify the type of support provided to HCPs to mitigate the negative impact of accreditation. It was difficult to define the measures adopted by managers to support HCPs during the accreditation process. However, many participants referred to teamwork and coworkers’ support to ease the effect of work demands during the preparation phase. While managers supported their employees during the preparation for accreditation, others experienced elevated stress. Therefore, HCPs could not seek psychological support from senior personnel. Ehlers et al. (2017) demonstrated that managers value positive external evaluations during accreditation, which might explain the level of tension in order to achieve such a positive assessment. Furthermore, strict features of management during the preparation phase were essential to engage workers in the preparation process as perceived by some HCPs.

This study did not intend to denounce healthcare accreditation; instead, it aimed to raise awareness of the consequences of psychosocial risks in healthcare accreditation. Findings showed that the process of accreditation increases work-related risks before the inspection visit. Some of the findings related to how accreditation processes increase psychosocial risks. These findings also consolidated the idea that appropriate systems and support for HCPs should be a priority when planning for accreditation. Furthermore, organizations should plan and inform HCPs for what to expect from the process of accreditation. While many healthcare organizations experience the challenging demands of accreditation, these organizations are required to prepare in advance for such inspection. Further, organizations need to develop a structured process for HCPs to balance between patients care and requirements of accreditation. According to this study, we can infer that the accreditation process has a clear impact on the psychosocial risks in healthcare facilities before the inspection visit. These findings raise intriguing questions about the nature and extent of accreditation regarding high job demands, inconsistent job resources, and unclear management practices to prioritize HCPs’ psychological health during the assessment. Future studies should consider a longitudinal design to investigate the job demands-resources model and highlight the role of healthcare facilities in improving the safety climate as a supportive measure for workers during the accreditation process.

## Limitations

Within the context of the current study, data were collected in participants’ workplaces hoping that they would feel comfortable and have control over the data collection process. The interviewer was a postgraduate researcher with prior assumptions regarding the process of accreditation. It is essential to note that the interviewer’s professional background and knowledge of accreditation could have impacted the findings and shaped conclusions drawn from this study. Since the study was limited to healthcare organizations in the United Arab Emirates, the results might not be relevant to other settings because of the cultural differences. The study, however, aimed to capture HCPs’ perceptions and experiences regarding accreditation, thereby adding to the existing literature. Another limitation is that the study relied on different recruitment methods that could have led to biased responses from participants. Therefore, the study could not rule out nonrandomized bias in the selected sample. However, the data provided rich information about the process of accreditation, which was consistent with the literature (Ho et al., 2014; Brubakk et al., 2015; Bogh et al., 2018). While the data collected in this study was comprehensive, a possible limitation of the interviews and focus groups might be the participants’ overreporting of negative perceptions about management support. Such perceptions could be due to the unclear relationship between HCPs and their managers, which underestimated the role of managers during accreditation. However, during interviews and focus group discussions, participants acknowledged the support provided by managers by means of simple words of encouragement, suggesting unclear support during the accreditation process.

## Conclusion

The impact of healthcare accreditation has been investigated widely over the past decades; however, there have been few published qualitative studies that focus on the apparent psychosocial risks in the context of healthcare accreditation processes. Most studies in this field have only focused on the outcome of accreditation as an opportunity to structure and organize the working environment. So far, however, there has been little discussion about the psychosocial risks that go hand-in-hand with the process of accreditation. This study mainly focused on emerging psychosocial risks during the implementation of healthcare accreditation. This study showed that the process of accreditation increases work-related risks before the inspection visit. Such risks were identified as increased job demands and work pace, conflicting information, and perceived strain. Furthermore, the supportive role of management was not clear or standardized during this process. These findings have significant implications for understanding how accreditation processes increase psychosocial risks; they also consolidate the idea that appropriate systems and support for HCPs should be a priority when planning for accreditation. A key policy priority should therefore be to plan for the long-term impact of psychosocial risks that may be associated with accreditation. Despite its limitations, this study adds to our understanding of the challenges and supports experienced by HCPs throughout the process of accreditation. These findings provide the following insights for future research. Further research using both qualitative and quantitative methods is needed to strengthen the findings related to the opportunities and threats accreditation poses to HCPs. Therefore, a greater focus on increasing the awareness of policy makers about the consequences of psychosocial risks could be useful in sustaining improvement initiatives in the healthcare sector.

## Data Availability Statement

All datasets generated for this study are included in the article/Supplementary Material.

## Ethics Statement

Ethical permissions were obtained from the Division of Psychiatry and Applied Psychology Research Ethics Sub-Committee at the University of Nottingham (i.e., Reference Number – 0236) and the Ethical Committee at the Ministry of Health and Prevention in the United Arab Emirates (MOHP/REC-40/2018). Participants were given information sheets that described the purpose of the study. After reading the information sheets, participants were asked to sign a consent form prior to commencing the talk. Written, informed consent was obtained from the individual for the publication of any potentially identifiable images or data included in this article.

## Author Contributions

AA contributed to the design of the study, the collection of the data, the development of the outcomes, and drafting of the manuscript. LT contributed to the design of the study, analysis of the data, and preparation of the manuscript. AS read and critically revised the outcomes. All the authors have read and approved the final manuscript.

## Disclaimer

Although the study was conducted in public healthcare organizations in the United Arab Emirates (UAE), the reported results do not reflect views of the Ministry of Health and Prevention in the UAE.

## Conflict of Interest

The authors declare that the research was conducted in the absence of any commercial or financial relationships that could be construed as a potential conflict of interest.

## Abbreviations

CQI: continuous quality improvement
HCPs: healthcare professionals
JD-R: Job Demand-Resources
REC: Research Ethical Committee
UAE: United Arab Emirates.
