# Supplementary material for: What Impact Does Accreditation Have on Workplaces? A Qualitative Study to Explore the Perceptions of Healthcare Professionals About the Process of Accreditation
Source: Front Psychol. 2020 Jul 10;11:1614. doi: 10.3389/fpsyg.2020.01614 (PMC7365862; doi:10.3389/fpsyg.2020.01614)
Supplement: Supplementary file 1 [file Table_1.docx]

Supplementary Material

Table 1 Descriptive numbers of participants in interviews and focus group discussions.

| Profession | Interview | Focus group | Total |
| --- | --- | --- | --- |
| Nurses | 14 | 6 | 20 |
| Doctors | 9 | 7 | 16 |
| Allied Health Professionals | 4 | 4 | 8 |
| Administrators | 0 | 5 | 5 |
