# Supplementary material for: What Impact Does Accreditation Have on Workplaces? A Qualitative Study to Explore the Perceptions of Healthcare Professionals About the Process of Accreditation
Source: Front Psychol. 2020 Jul 10;11:1614. doi: 10.3389/fpsyg.2020.01614 (PMC7365862; doi:10.3389/fpsyg.2020.01614)
Supplement: Supplementary file 2 [file Table_2.docx]

Supplementary Material

Table 2 Interviews and focus groups question scheme.

| Questions Scheme |
| --- |
| 1. Can you tell me what do you do for a living?    1. Can you describe your profession?    2. For how long were you working here? 2. I would like to start out by just getting to understand your feelings toward accreditation.    1. What happened so that your facility achieved accreditation?    2. Can you tell me more about your emotional and mental states to prepare for accreditation? 3. Let’s hear about the changes that occur at your work:    1. What duties does accreditation add to your typical work?    2. Can you describe the speed of work at your unit while preparing for accreditation?    3. Has your work always been that way; if it has changed, can you tell me more when did that happen?    4. How do you feel about those changes?    5. Can you think of any ways that these changes might affect the actual care that the patients received?    6. How do your colleagues think of work changes in accreditation? 4. Can you think of any potential issues that could risk your health? 5. Would you please tell me about any of the changes in your work that created risks of injuries mentally and physically?    1. How did those injuries affect you?    2. What did you do after you injured yourself? 6. Have there been any efforts to improve the emotional and mental health of healthcare providers with these changes?    1. How does your leader/supervisor recognize problems that affect healthcare providers’ emotional and mental well-being?    2. Can you give me an example? 7. How does your facility provide support that helps reduce side effects of work demands? 8. Would you describe what happens after an employee contributes or gives suggestions that address workers’ mental and emotional safety? 9. Do you have additional thoughts about whether policies and procedures would be enough to promote workers’ mental, emotional, and social health? 10. Before we end, I was just wondering if there is any other feedback you would like to provide about the accreditation process and how you would like to do it differently. 11. What are the negative and positive aspects of change you have noticed while preparing for accreditation? 12. How did the preparation phase influence your normal or personal life? |
