# Supplementary material for: What Impact Does Accreditation Have on Workplaces? A Qualitative Study to Explore the Perceptions of Healthcare Professionals About the Process of Accreditation
Source: Front Psychol. 2020 Jul 10;11:1614. doi: 10.3389/fpsyg.2020.01614 (PMC7365862; doi:10.3389/fpsyg.2020.01614)
Supplement: Supplementary file 3 [file Table_3.docx]

Supplementary Material

Table 3 Themes and Sub-themes.

|  | Theme | Sub-theme | Codes |
| --- | --- | --- | --- |
| **1** | **Challenging Factors in the Process of Accreditation** | Focused efforts on administrative “work” | Participating in Additional Meetings |
|  |  |  | Reading Policies |
|  |  |  | Training and Educational Activities |
|  |  |  | Focusing Patient Documentation and Record-Keeping |
|  |  | Observed work-related risks | Time Pressure due to Delays |
|  |  |  | Working Additional Hours |
|  |  |  | Increased Work Pace |
|  |  |  | Conflicting Information |
|  |  | Perceived Pressure of Accreditation | Impact on Physical and Emotional Health |
|  |  |  | Impact on Family and Social Life |
|  |  | Managements’ role during accreditation | Perceived Lack of Managers Support / Stress Managers |
|  |  |  | Fear of Being Blamed |
|  |  |  | Restrictions on leaves |
| **2** | **Enablers to Achieve Accreditation** | Supportive approaches in accreditation | Observed Collaboration and Teamwork |
|  |  |  | Support and Encouragement from Managers |
|  |  |  | Recognition of Achievement |
|  |  | Meaningful work after accreditation | Meaningful work |
|  |  |  | Organized Working Environment |
